# Sensitivity Analysis of Parameters Affecting Wetland Water Levels: A Study of Flood Detention Basin, Colombo, Sri Lanka

**DOI:** 10.3390/s23073680

**Published:** 2023-04-02

**Authors:** Madhawa Herath, Tharaka Jayathilaka, Hazi Mohammad Azamathulla, Vishwanadham Mandala, Namal Rathnayake, Upaka Rathnayake

**Affiliations:** 1 Department of Mechanical Engineering, Faculty of Engineering, Sri Lanka Institute of Information Technology, Malabe 10115, Sri Lanka; 2 Department of Civil Engineering, Faculty of Engineering, Sri Lanka Institute of Information Technology, Malabe 10115, Sri Lanka; 3 Department of Civil Engineering, Faculty of Engineering, University of the West Indies, St. Augustine P.O. Box 331310, Trinidad and Tobago; 4 Department of computer science, Indiana University, Bloomington, IN 47405, USA; 5 School of Systems Engineering, Kochi University of Technology, Tosayamada 782-8502, Japan; 6 Department of Civil Engineering and Construction, Faculty of Engineering and Design, Atlantic Technological University, F91 YW50 Sligo, Ireland

**Keywords:** artificial neural networks (ANN), Colombo flood detention basin, meteorological parameters, sensitivity analysis, water levels, wetlands

## Abstract

Wetlands play a vital role in ecosystems. They help in flood accumulation, water purification, groundwater recharge, shoreline stabilization, provision of habitats for flora and fauna, and facilitation of recreation activities. Although wetlands are hot spots of biodiversity, they are one of the most endangered ecosystems on the Earth. This is not only due to anthropogenic activities but also due to changing climate. Many studies can be found in the literature to understand the water levels of wetlands with respect to the climate; however, there is a lack of identification of the major meteorological parameters affecting the water levels, which are much localized. Therefore, this study, for the first time in Sri Lanka, was carried out to understand the most important parameters affecting the water depth of the Colombo flood detention basin. The temporal behavior of water level fluctuations was tested among various combinations of hydro-meteorological parameters with the help of Artificial Neural Networks (ANN). As expected, rainfall was found to be the most impacting parameter; however, apart from that, some interesting combinations of meteorological parameters were found as the second layer of impacting parameters. The rainfall–nighttime relative humidity, rainfall–evaporation, daytime relative humidity–evaporation, and rainfall–nighttime relative humidity–evaporation combinations were highly impactful toward the water level fluctuations. The findings of this study help to sustainably manage the available wetlands in Colombo, Sri Lanka. In addition, the study emphasizes the importance of high-resolution on-site data availability for higher prediction accuracy.

## 1. Introduction

Wetlands are globally diverse ecosystems found in all climate zones, from the tropics to the tundra [1]. They cover approximately 6% of the Earth’s surface [2]. Wetlands have been defined in several ways, depending on their functional characteristics, purpose, and geographic context [3]. According to Ramsar convention, ‘wetlands are areas of marsh, fen, peatland or water, whether natural or artificial, permanent or temporary, with water that is static or flowing, fresh, brackish or salt, including areas of marine water the depth of which at low tide does not exceed six meters. Wetlands play an integral role in environmental, social, and economic aspects [4]. They regulate the global climate, maintain the hydrological cycle, protect ecosystem diversity, and maintain human well-being [5]. Wetlands are considered nature’s supermarkets as they support a diverse food chain [6]. Despite being vital components of the global ecosystem, wetlands are facing continuous threats due to rapid developments in infrastructure and agriculture [7]. On the other hand, degradation of wetland special coverage takes place due to natural circumstances, such as invasive species, etc. [8]. ‘Global Wetland Outlook 2021’ reports that global wetland special coverage has been drastically reduced by 35% since 1970 [9]. Therefore, it is vital to identify the threats to the wetlands and take the necessary actions to protect these most productive ecosystems.

Wetland hydrology is one of the major concerns in proper wetland operation. In addition, the water level is the primary factor controlling the structure and function of the wetlands [10]. Wetland water levels vary tremendously depending on the time and duration of surface water inundation as well as seasonal patterns of inundation [11,12]. The dominant factors that affect wetland water levels are precipitation, evaporation, temperature, wind speed, humidity, upstream water inflows, downstream water outflows, and groundwater flow [13,14,15]. Furthermore, changes in land use or drainage of wetland catchments can thus disrupt the natural balance of wetland hydrology [16]. Measuring and predicting wetland water levels are important for several reasons. The most common method to determine the wetland water levels is to compute water balance based on the above-mentioned parameters. However, obtaining a precise water balance is challenging in most scenarios. Water level measurements of the wetlands are limited in most countries, including Sri Lanka. Furthermore, available data could have some distractions as they are not measured in the field simultaneously [17]. This study focuses on evaluating the relative importance of each hydro-climatic parameter with respect to the wetland water level fluctuations.

Only a few studies can be found in the literature on the sensitivity analysis of hydro-meteorological data with respect to wetland water level predictions. Dadaser-Celik and Cengiz [18] performed a sensitivity analysis for Sultan marshes in Turkey, using six configurations by excluding single input parameters. They found that Sultan marsh’s water level was highly dependent on the previous month’s water level with respect to the other inputs. The least related parameters were found to be air temperature and evaporation. Atashi et al. [19] conducted a time series analysis for the Red River of the north-central United States and central Canada using Seasonal Autoregressive Integrated Moving Average (SARIMA), Random Forest (RF), and Long Short-Term Memory (LSTM). They used hourly water level data to evaluate the water levels six hours, twelve hours, one day, three days, and one week in advance. The results have shown that the LSTM method outperforms the other two methods. In addition, Choi et al. [20] developed a wetland water level prediction model using ANN, Decision Tree (DT), Random Forest (RF), and Support Vector Machines (SVM) to simulate the water levels in the Upo wetland, South Korea. They suggested considering the downstream water flow as one of the inputs for the model development as they found that to be an important parameter.

Furthermore, Karthikeyan et al. [21] conducted a study to evaluate the performance of neural networks in simulating and forecasting groundwater levels of a coastal wetland in India. They compared two neural network architectures, Feed Forward Neural Network (FFNN) and Recurrent Neural Network (RNN), trained under five algorithms, namely the Levenberg Marquardt (LM) algorithm, the Resilient Back propagation algorithm, the BFGS Quasi-Newton algorithm, the Scaled Conjugate Gradient (SCG) algorithm, and the Fletcher Reeves Conjugate Gradient algorithm. According to their results, FFNN trained with the Fletcher Reeves Conjugate Gradient algorithm outperformed all other combinations. Altunkaynak [22] also carried out a study in Lake Van in Turkey to develop a neural network model to predict the water level variations. In their study, model training was carried out using a back propagation algorithm. They discovered that neural networks produce accurate results, although the relationships among the parameters are more complex.

According to the literature, a limited number of studies have been carried out on wetland water level predictions and on the sensitivity of affecting parameters. Most of the studies have investigated river or lake water level fluctuations. Furthermore, there are some differences in the geo-hydro parameters of the wetlands compared to the rivers and lakes. On the other hand, the South Asian region lacks recent studies on wetland water level predictions. As per the authors’ knowledge, the first attempt in the context of Sri Lanka to develop a water level simulation model was initiated by Jayathilake et al. [23] as an initial attempt. It investigated the applicability of ANNs to predict the water levels in a critical wetland in Colombo, Sri Lanka. However, a sensitivity analysis of factors affecting wetland water levels was never tested in the context of Sri Lanka. Therefore, to address that research gap, we have focused on finding the relative importance of each meteorological parameter on wetland water level fluctuations. The modelling approach used in this study provides a convenient tool for determining the impact of hydro-climatic processes on wetland water levels. Furthermore, the results of the sensitivity analysis would provide a better understanding of the importance of each parameter, so that future studies can focus more on those parameters. This will also be more cost-effective when collecting the data for future studies.

The Colombo flood detention wetland area was selected as the testing site for the analysis. Colombo is the capital of Sri Lanka, and some of the important areas are built upon the marshy lands. Due to urbanization, wetland degradation in Colombo is relatively high. As a result of that, frequent floods are more common in Colombo city as it has reduced the flood-holding capacity of the wetlands. Therefore, it is important to protect and manage the wetlands in Colombo city. In addition, it has been noticed that less emphasis is given to monitoring and forecasting water levels in Sri Lankan wetlands. Therefore, the proposed study would be useful in achieving the sustainability of the wetland ecosystems in the city of Colombo, Sri Lanka.

## 2. Materials and Methodology

This section presents the mathematical formulation of the sensitivity analysis problem, identification of environmental factors (i.e., variables), preprocessing of input data, selection and modification of neural network architectures, performance measuring indices, and the hierarchical strategy of selecting optimal input variable combinations for wetland water level prediction.

**Figure 1:**
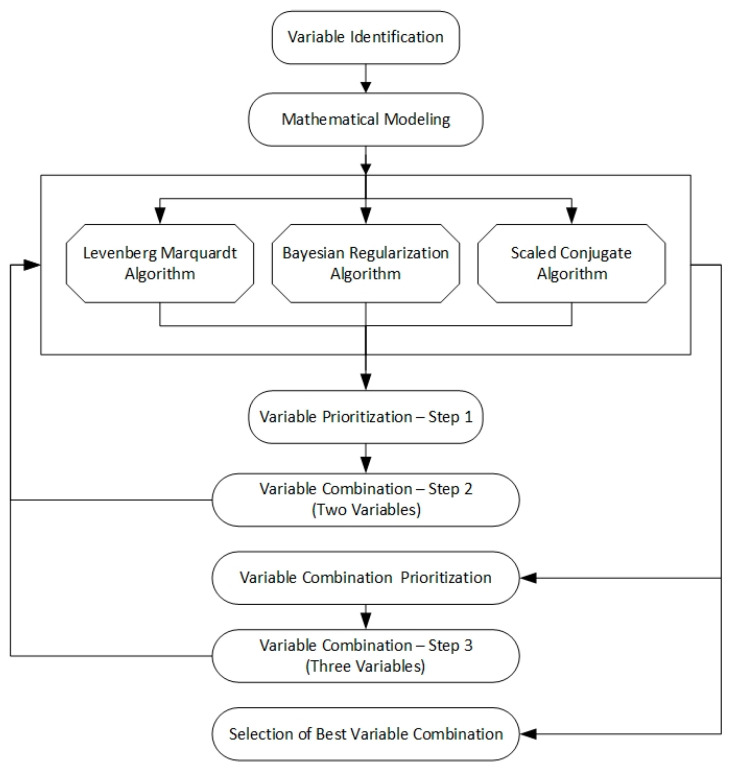
Schematic diagram of the methodology.

As per the extent literature, seven key factors have been identified, including daily precipitation to the catchment, minimum daily temperature, maximum daily temperature, daily relative humidity during the daytime, daily relative humidity at nighttime, daily evaporation, and daily average wind speed, as the independent variables that the wetland water level depends on. These parameters directly and indirectly help in balancing the hydrological cycle, which impacts the wetland water levels. Higher coefficients of determination (R^2^) can be found in many research studies for the relationship between those independent variations to wetland water levels [23]. This shows a strong relationship between these variables and the wetland water level. Therefore, this study has been proposed to identify the most sensitive independent variables to the fluctuations of wetland water level and their most optimal combination, which can be used to predict the wetland water level. The study adopted a neural network as the mapping function between the independent variable(s) and water level, considering easy and accurate implementation with minimum human intervention. However, the sensitivity of each variable to the wetland water level can vary, and the methodology illustrated in Figure 1 was adopted to find the most sensitive independent variable, along with the optimal variable combination.

### 2.1. Data Preprocessing

The accuracy of the NN model prediction and the time taken for model convergence heavily depends on the quality of training data. Usually, it contains many errors within the time series sensor data due to technical faults as well as human errors. Therefore, data preprocessing before feeding data into NN is essential. Some of the key issues are asynchronous data fields, missing data points, spikes (i.e., outliers), and data duplication contained in the training data. Hence, the following preprocessing steps were adopted.

1. First, all data fields, including water level and independent variables, were rearranged to ascending time stamps.
2. Then, each data field was analyzed in detail for missing data points. If the missing data lengths were less than 3 days, they were forward filled. If not, the missing data points were removed completely from the other data fields.
3. Next, the missing data clusters with error data points were identified and removed from the dataset using manually identified threshold values for each field.
4. Independent variable readings were spread in different ranges in varying amplitudes. For example, the temperature fluctuates between 20 and 35 °C, the RH value is between 65 and 95%, the evaporation is between 2 and 3 mm, and the water level is between 0.2 and 0.4 m. This variation adversely affects the NN training and time taken for model convergence. Therefore, all data fields that contain recorded meteorological parameters were normalized separately, as presented in Equation (1).

(1) $${\bar{y}}_{t}=\frac{\left(y_{t}-y^{min}\right)}{\left(y^{max}-y^{min}\right)}$$

where $y_{t}$ is the independent variable value, ${\bar{y}}_{t}$ is the normalized value, $y^{min}\;$is the minimum value, which is equal to zero in some of our data series, and $y^{max}$ is the maximum value of the data series. Due to limited data volume, the complete data set was fed into NN without the preparation of mini-batches.

### 2.2. Mathematical Modeling

As Figure 1 illustrates, initially the relationship between each independent variable and the wetland water level was investigated in Step 1. A Neural Network was used to represent the mapping function among the independent variable and the water level, as presented in Equation (2).

(2) $$WL=F_{\theta_{i}}\left(V_{i}\right)+\delta_{i}$$

where *WL* is the wetland water level, $V_{i}$ is the $i^{th}$ independent variable, and $F_{\theta_{i}}$ and $\delta_{i}$ are the mapping function and possible error for the $i^{th}$ independent variable, respectively. After comparing the relative sensitivity (i.e., R^2^ score and mean squared error (MSE)) of each independent variable, the most sensitive variables were identified. Thereafter, Step 2 selected highly sensitive independent variables, which were grouped as two variables with all possible combinations for further analysis for optimal bi-variable combination. The bi-variable mapping function is presented in Equation (3).

(3) $$WL=F_{\theta_{i},j}\left(V_{i},V_{j}\right)+\delta_{i,j}$$

where $V_{i}$ and $V_{j}$ are the $i^{th}$ and $j^{th}$ independent variables and $F_{\theta_{i,j}}$ and $\delta_{i,j}$ are the mapping function and possible error for $i^{th}$ and $j^{th}$ independent variables. The R^2^ and MSE value for each combination were analyzed, and the most optimal three combinations were identified. Thereafter, the investigation was repeated for tri-variable combinations in Step 3. The tri-variable mapping function is presented in Equation (4).

(4) $$WL=F_{\theta_{i},j,k}\left(V_{i},V_{j},V_{k}\right)+\delta_{i,j,k}$$

where $V_{i}$, $V_{j}$, and $V_{k}$ are the $i^{th}$, $j^{th}$, and $k^{th}$ independent variables, respectively. $F_{\theta_{i,j,k}}$ and $\delta_{i,j,k}$ are the mapping function and possible error for $i^{th}$, $j^{th}$, and $k^{th}$ independent variables.

### 2.3. Neural Network Modeling

The relationships between time series data (i.e., wetland water level) and environmental parameters are highly nonlinear [24,25]. Hence, the development of traditional mathematical mapping functions is very difficult, and manual selection of the most optimal feature vector for the given problem is almost impossible. Because NNs are highly capable of self-learning and selecting the most optimal feature vector for the assigned function automatically, the authors decided to select an inbuilt NN model in MATLAB for the analysis.

A “non-linear input-output network” NN model was selected, which can keep the memory of previous input data for future prediction for the analysis. Retaining information related to previous input will enhance the accuracy of prediction for time series data such as wetland water levels. The selected NN uses the Sigmoid activation function at the hidden layers to handle the non-linearity of the problem and the Liner activation function at the output layer neuron for predicting the wetland water level as a real value. There are three main types of optimization algorithms used for NN training, including the Levenberg Marquardt algorithm, the Bayesian Regularization algorithm, and the Scaled Conjugate algorithm. Those optimization algorithms can present varying degrees of biases for different independent variable–water level relationships. Hence, in order to conduct an unbiased sensitivity analysis on a common platform, all three algorithms were used for all input–output relationship models separately and calculated the ensemble average of R^2^ and MSE. Similarly, for Step 2 and Step 3, all the mapping relationship sensitivities were analyzed with respect to the three different optimization algorithms. The selected optimization algorithms behave in different ways, as explained in the following sections.

#### 2.3.1. Levenberg Marquardt Algorithm (LM)

The Levenberg Marquardt algorithm combines the steepest descent algorithm and the Gauss-Newton algorithm [26]. It reduces the sum of square error functions to a minimum. Therefore, the Levenberg Marquardt algorithm is used to solve the non-linear least squares problems [27]. It is one of the fastest learning algorithms and requires more memory, but less time [28]. Many software applications use the Levenberg Marquardt algorithm to solve generic curve-fitting problems [29]. This algorithm is suitable for small- and medium-sized problems [30]. The Levenberg Marquardt algorithm can be expressed as Equation (5).

(5) $$x_{k+1}=x_{k}-{\left[\;J^{T}\;J+\mu \;I\;\right]}^{-1}\;\;J^{T}e$$

where *x* is the input variable, *J* is the Jacobian matrix of the performance criteria to be minimized, *µ* is the coefficient of combination (which is always a positive value and controls the learning process of the algorithm), *I* is the identify matrix, *k* is the iteration index, and *e* is the residual error vector. In addition, *T* stands for transposition.

#### 2.3.2. Bayesian Regularization Algorithm (BR)

In the Bayesian neural network algorithm, weights are considered a probability distribution that estimates the uncertainty in weights and predictions [31]. This probability distribution is mathematically given in Equation (6).

(6) $$P\;(parameters|data)=\frac{P\;(data|parameters)\times P\;\left(parameters\right)}{P\;\left(data\right)}\times likelihood\times prior$$

where P (parameters|data) is the likelihood and P (parameters) is the prior distribution. There are some advantages of Bayesian neural networks compared to the other methods. They are more robust, and generalization is comparatively better [32]. They quantify the uncertainty and are used in many practical applications. These networks provide solutions to a variety of QSAR modeling problems, including model selection, model robustness, validation set selection, validation effort size, and neural network architecture optimization [33]. Furthermore, the Bayesian Regularization algorithm is difficult to overfit as it calculates and trains using a limited number of effective network parameters [34].

#### 2.3.3. Scaled Conjugate Gradient Algorithm (SCG)

The Scaled Conjugate Gradient algorithm is a second order conjugate algorithm where the weights are in the direction where the performance function is dropping rapidly [35]. It is a supervised learning algorithm and is generally used to solve large-scale problems. This method has a step-size scaling method that eliminates the need for time per learning iteration. [36]. The SCG algorithm is comparatively inexpensive in terms of the required processing power and memory because it does not follow a traditional line search in each iteration. Most of the wetland water level prediction applications need to be installed remotely when a center server connectivity is not possible. Therefore, the processing algorithm should be lightweight. However, one disadvantage of this method is increased learning time [37]. The Scaled Conjugate Gradient algorithm can be expressed as Equation (7).

(7) $$X_{K}=X_{K-1}+\alpha_{K}d_{K-1}$$

where *X* is the input variable, $\alpha_{K}$ is the step length at *k*th iterations, $d_{K}$ is the search direction, and *k* is the iteration index. As explained above, only three neural network optimization algorithms were used in this study. Several other algorithms, including Newton fitting technique-based algorithms [38] and Newton iteration-based algorithms and their variants [39] could also be used in future studies.

### 2.4. Performance Evaluation of the NN Model

The performance of the sensitivity analysis was evacuated using two key indices: coefficient of determination (R^2^) and mean squared error (MSE). The R^2^ usually evaluates the relationship between actual and predicted values. However, in addition to the R^2^ values, the study used R^2^ graphs to present the relationship between actual and predicted wetland water levels by the NN model.

#### 2.4.1. Coefficient of Correlation (R^2^)

The Coefficient of correlation is a statistical indicator that illustrates the strength of linear dependence between two variables [40]. It represents the proportion of the variance for a dependent parameter, which is illustrated by an independent parameter in a regression model [41]. Whereas correlation describes the strength of the relationship between an independent and dependent variable, R-squared describes the extent to which the variance of one variable explains the variance of the second variable. It can be represented as the following Equation (8).

(8) $$R^{2}=\frac{\sum_{i=1}^{N}\left(X_{i}-\bar{X}\right)\left(Y_{i}-\bar{Y}\right)}{\sqrt{\sum_{i=1}^{N}{\left(X_{i}-\bar{X}\right)}^{2}\;}\;\sqrt{\sum_{i=1}^{N}{\left(Y_{i}-\bar{Y}\right)}^{2}\;}}$$

where $Y_{i\;}$ indicates the observed water level and $X_{i}$ indicates the predicted water level. According to the above equation, if the R^2^ is close to 1, this indicates a close relationship between the variables, and when it is close to zero, this indicates a poor relationship between the variables [42]. In addition, adjusted R^2^ was also tested for the relationships. The adjusted R^2^ takes into account the number of independent variables used for predicting the target variable and increases when the new term improves the model more than would be expected by chance.

#### 2.4.2. Mean Squared Error (MSE)

Mean squared error illustrates the difference between the observed and predicted values [43]. MSE is a risk function, corresponding to the expected value of the squared error loss. It is always a positive value. A model performs better when the MSE values are close to zero. Zero MSE indicates no errors in the model. MSE can be expressed as the following Equation (9).

(9) $$\mathrm{MSE}=\frac{1}{N}\;\overset{N}{\underset{i=1}{\sum }}{\left(Y_{i,observed}-Y_{i,predicted}\right)}^{2}$$

## 3. Case Study

**Figure 2:**
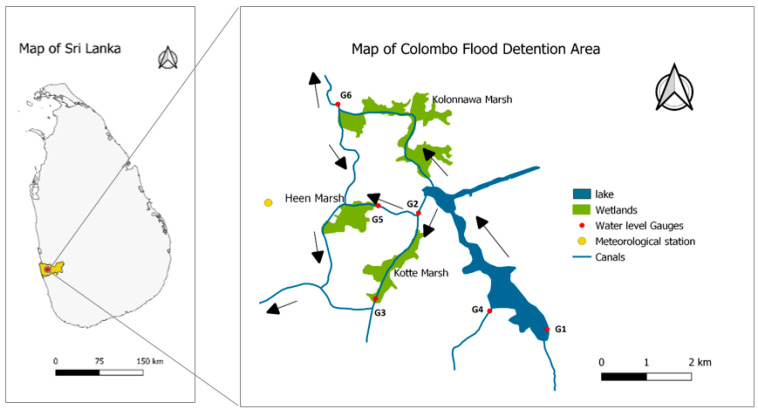
Map of Colombo flood detention basin.

The developed NN model was then applied to the Colombo flood detention basin in Sri Lanka, which is in the western province of Sri Lanka (7°0′0″ N and 79°50′15″ E to 7°0′0″ N and 80°0′0″ E). The altitude of this area is, on average, 1 m above mean sea level. The spatial coverage is approximately 400 ha (Kolonnawa marsh = 214.3 ha; Kotte marsh = 97.4 ha; Heen marsh = 87.7 ha). Three separate wetlands, including Kimbulawala, Kotte Canal, and Kotte North wetland were selected for the analysis to take a more generalized approach by omitting any wetland-specific relationships. However, these marshes are interconnected and play a crucial role in flood accumulation within Colombo city. The Colombo flood detention area is located within the wet zone and has an average annual rainfall of 2000–2300 mm and an average annual temperature ranging from 25 to 27 °C. Figure 2 illustrates the map of the Colombo flood detention area. The water level gauges (G1–G6) are shown by the red dots, whereas the Colombo meteorological station is shown by beige dot. The arrows show the flow directions and the connectivity among the three wetlands.

Colombo city can be identified as a city built on and around wetlands. It is extremely vulnerable to flash floods due to heavy rainfall. A recent study has shown that the Colombo flood basin capacity has been reduced by 30% as a result of various infrastructure developments [44]. Further, it shows that the wetland loss rate in Colombo city is approximately 1.2% per annum. Hence, flood management of the Colombo region could be addressed by implementing measures to manage and safeguard the Colombo wetlands. Other than the flood risks, there are many other severe results due to the degradation of the Colombo flood detention area, such as poor water quality, threats to the native flora and fauna, bad influence on the well-being of urban poor, etc.

The water level data were not documented properly due to various logistic reasons, and therefore the authors obtained the daily data from 2004 to 2012 from the Land Development Corporation, Sri Lanka. To match the time duration, the daily precipitation to the catchment, minimum daily temperature, maximum daily temperature, daily relative humidity during the daytime, daily relative humidity at nighttime, daily evaporation, and daily average wind speed were purchased from the Department of Meteorology, Sri Lanka. However, the analysis would have been much stronger with higher-resolution hourly data. In addition, on-site meteorological data would give a better understanding of the natural processes. Therefore, this research has been carried out with the above-stated limitation of data. Nevertheless, the research emphasizes the importance of having rich databases that use sensors.

As described in the methodology section, the analysis was carried out with the available hydro-meteorological parameters using three algorithms. The neural network modeling, training, and testing were conducted on a personal computer with an Intel^®^ Core i7 processor with 32GB RAM and Nvidia^®^ GPU with 2GB memory.

## 4. Results and Discussion

### 4.1. Sensitivity of Single Variable

The sensitivity of each independent variable (and combinations of variables) in predicting wetland water level has been measured as R^2^ and MSE values of the testing phase of the respective NN. Table 1 presents the R^2^ and MSE values of each independent variable for the three wetlands considered for the analysis under three optimization algorithms.

**Figure 3:**
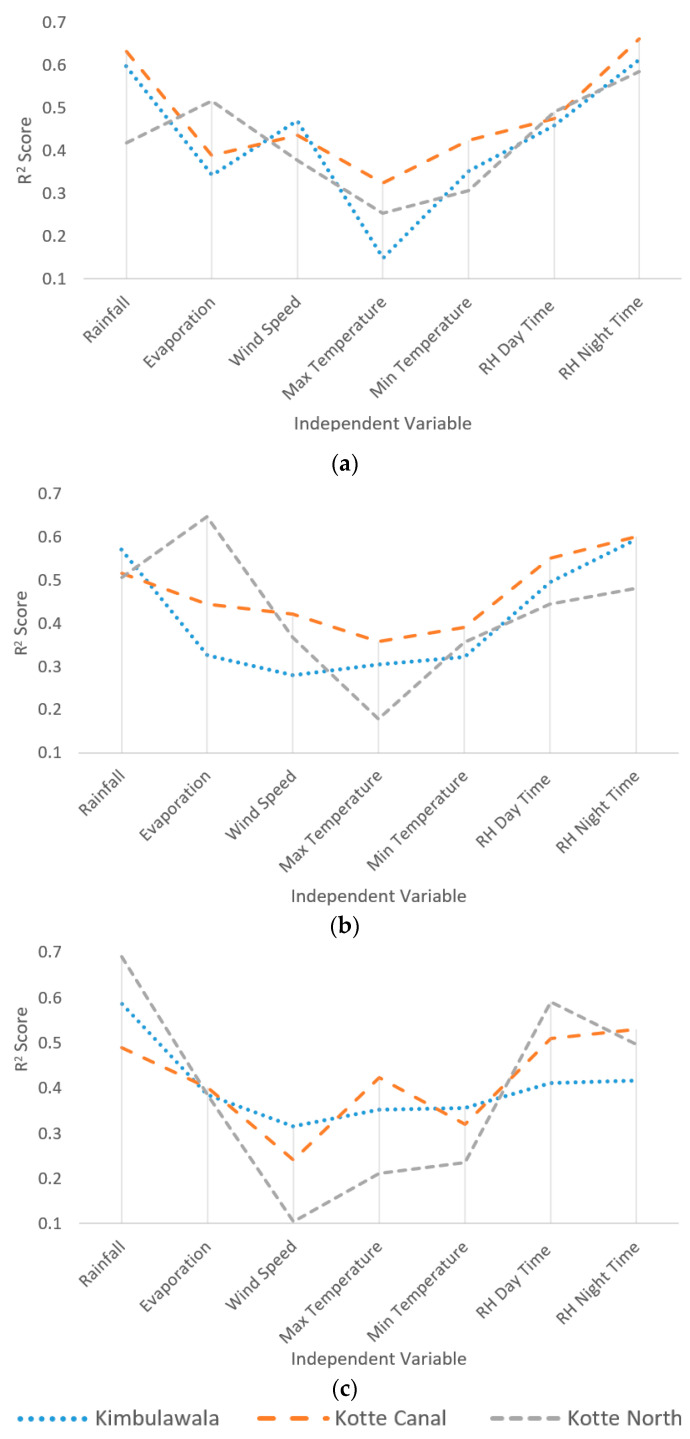
Sensitivity Analysis (R^2^ Score): (**a**) under the Levenberg Marquardt algorithm; (**b**) under the Bayesian Regularization algorithm; (**c**) under the Scaled Conjugate Gradient algorithm.

As per the results presented in Table 1, rainfall and daytime relative humidity presented a strong relationship for water level fluctuation. Furthermore, relative humidity at nighttime also presented a significant relationship with all wetland water levels. The relationship between the rainfall in the catchment and the wetland water level is quite obvious. However, having relationships to relative humidity over other meteorological parameters is interesting. Detailed analyses are showed in Figure 3a–c. They present the variations of relationships between individual parameters.

As per the results presented in Figure 3a,b, Kimbulawala and Kotte Canal wetlands behave in a similar pattern in all optimization algorithms. However, the Kotte North wetland behaves in a slightly different pattern from the other two wetland, especially with the Levenberg Marquardt and Bayesian Regularization algorithms. Nevertheless, it showed a similar behavior to other wetlands for the Scaled Conjugate Gradient algorithm. Therefore, it can be concluded that all three wetlands presented a considerably similar relationship pattern between the independent variables considered for the analysis and wetland water levels. Hence, the results are more generalized rather than wetland specific in nature.

The most sensitive factor for water level prediction is rainfall, which is explicitly explainable. It is obvious that with rainfall the water level of the wetland can increase. However, most of the days were not rainy days, yet the relationship still exists. The relative humidity also presented a strong relationship to water level variation, especially in the nighttime. All the investigated wetlands are situated within Colombo in closer proximity to the ocean. Sri Lanka is a tropical country, and it gets direct sunlight throughout the day. Therefore, in the daytime, the air closer to the ground level is heated and lifted. The nighttime temperature of the ocean near the land is higher than the temperature of the land. As a result, the air will flow from the ocean to land with rich water vapor, which will lead to an increase the humidity. Hence, the nighttime humidity is much higher than the daytime humidity in the selected area. Once relative humidity is high, the evaporation goes down, and this may reduce the change (i.e., drop) in wetland water level.

Furthermore, evaporation also presented a considerable relationship with wetland water level, especially according to the Scaled Conjugate Gradian optimization algorithm; when the evaporation is higher, the impact on the water level is high. High evaporation leads to water level drop in the wetland. However, the temperature and wind speed have not presented strong relationships to the water level prediction. Usually the temperature difference (maximum and minimum) of a day is very small and in the range of 8–10 °C, and due to the high relative humidity, the impact of temperature on the evaporation and water level change is minimal. Moreover, for the same reason, when the air humidity is high, the impact of wind speed on the evaporation and water level is also low. Therefore, the given results by the analysis can be accepted with proper justifications.

**Figure 4:**
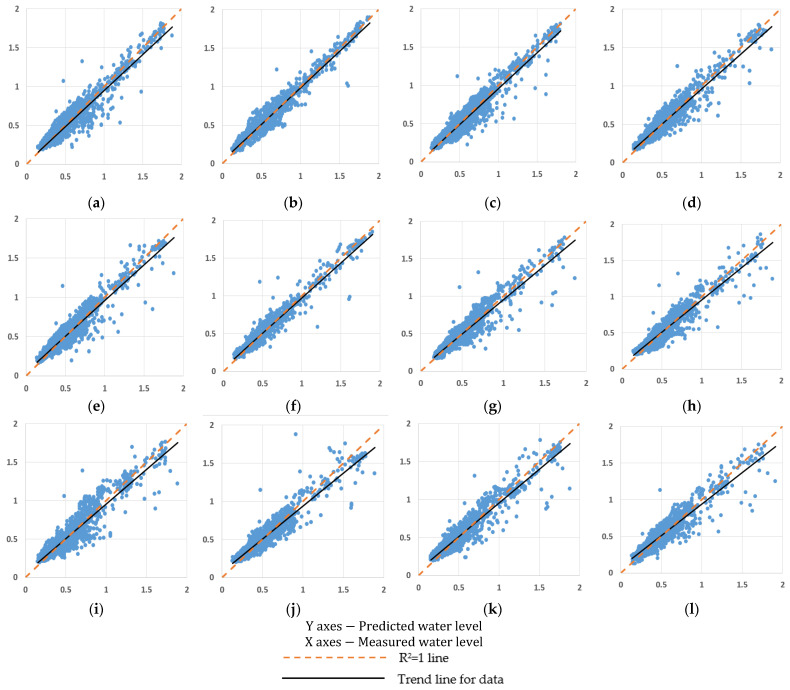
R^2^ Plots of Kimbulawala Wetland Water Level Prediction: (**a**) under Evaporation for BR; (**b**) under Rainfall for BR; (**c**) under Relative humidity at daytime for BR; (**d**) under Relative humidity at nighttime for BR; (**e**) under Evaporation for LM; (**f**) under Rainfall for LM; (**g**) under Relative humidity at daytime for LM; (**h**) under Relative humidity at nighttime for LM; (**i**) under Evaporation for SCG; (**j**) under Rainfall for SCG; (**k**) under Relative humidity at daytime for SCG; (**l**) under Relative humidity at nighttime for SCG.

The predicted wetland water levels against the measured water levels for the Kimbulawala wetland with respect to different independent variables under three algorithms are given in Figure 4a–l. The predicted values against the measured water levels show some possible deviations. The nonlinearity of the natural system may have impacted these results. However, the coefficient of determination values for each case aligned closer to 1. However, they do not clearly show the most important or sensitive parameter of the analysis. In addition, the two wetlands showed a similar pattern in predicted and measured wetland water levels.

### 4.2. Sensitivity of Bi-Variables

As the second step, the selected highly sensitive independent variables were grouped as pairs in all possible combinations (i.e., bi-variable) and tested for collective sensitivity for water level under the three optimization algorithms used for the previous analysis. The results of the bi-variable sensitivity analysis are presented in Table 2. The sensitivity was calculated as the R^2^ Score and MSE value of the testing phase of selected NN.

As per the results presented in Table 2, it can be seen that the independent variable combinations that include rainfall presented high sensitivity for wetland water level changes. For further analysis, the obtained sensitivity results are presented in Figure 4a–c.

As per the obtained results, the combinations that include rainfall presented a higher sensitivity in predicting wetland water level. However, compared to Step 1, most of the bi-variable combinations presented fairly limited variation (i.e., between 0.5 and 0.8) for all combinations. This implies that the behavior of a bi-variable combination is substantially different from the way individual variables behave. Hence, finding the optimal independent variable combination will not be a straightforward approach. As an example, the sensitivity of the relative humidity of daytime and evaporation for water level prediction is lower than other independent variables, such as rainfall and the relative humidity at nighttime. However, their combination performances are comparable to other variable combinations of rainfall and relative humidity at nighttime. This implies that the hidden relationships between individual variables are very complex, and NN was able to understand most of them within the current study.

Although the individual variables were considered as independent at the beginning of the study, the results of the Step 2 analysis present a considerable correlation between them. Therefore, the contribution of one variable for water level prediction can be fully or partially fulfilled by another variable. Hence, large scale distributed wetland water level prediction systems are required to conduct a detailed investigation to find location-specific optimal variable combinations. Moreover, the identification of the most sensitive variables will make the solution operationally less complex and cost effective.

**Figure 5:**
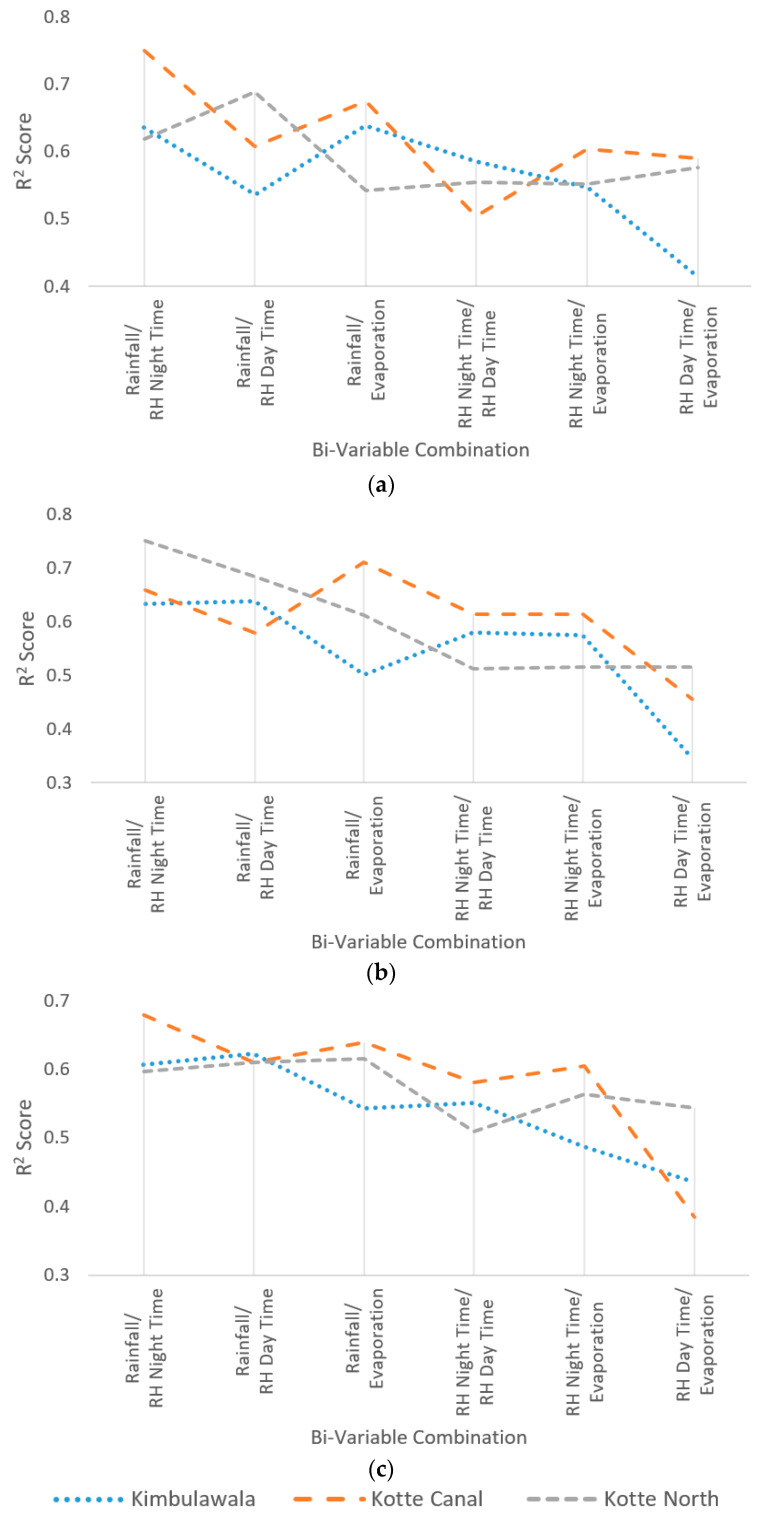
Bi-Variable Sensitivity Analysis (R^2^ Score): (**a**) under the Levenberg Marquardt algorithm; (**b**) under the Bayesian Regularization algorithm; (**c**) under the Scaled Conjugate Gradient algorithm.

As per Figure 5a–c, the combinations of rainfall and relative humidity of nighttime and rainfall and evaporation made the height contribution the combined variable for the wetland water level prediction. Moreover, all wetlands using the three optimization algorithms presented similar behavior, confirming the unbiasedness of the optimization algorithms and generalization of the achieved results without being specific to a given wetland.

### 4.3. Sensitivity of Tri-Variables

In Step 3, the selected most sensitive variables from Step 1 and combinations (bi-variable) from Step 2 for wetland water level estimation were grouped into several combinations (tri-variable), which included three variables. The selected combinations and their sensitivity, as measured by R^2^ Score and MSE value of the NN testing phase, are presented in Table 3.

As per the results, all three combinations presented a substantially higher sensitivity towards wetland water levels. Although rainfall, relative humidity during the daytime, and relative humidity at nighttime individually presented a high sensitivity, among the tri-variable combinations, the rainfall/relative humidity at nighttime/evaporation combination presented the highest sensitivity among other tri-variable combinations. However, among the bi-variable combinations, rainfall/relative humidity at nighttime and rainfall/evaporation presented the highest sensitivity, which justifies the selected most sensitive tri-variable combination. Figure 5a–c presents the tri-variable performance under the three optimization algorithms used for the NN training phase.

**Figure 6:**
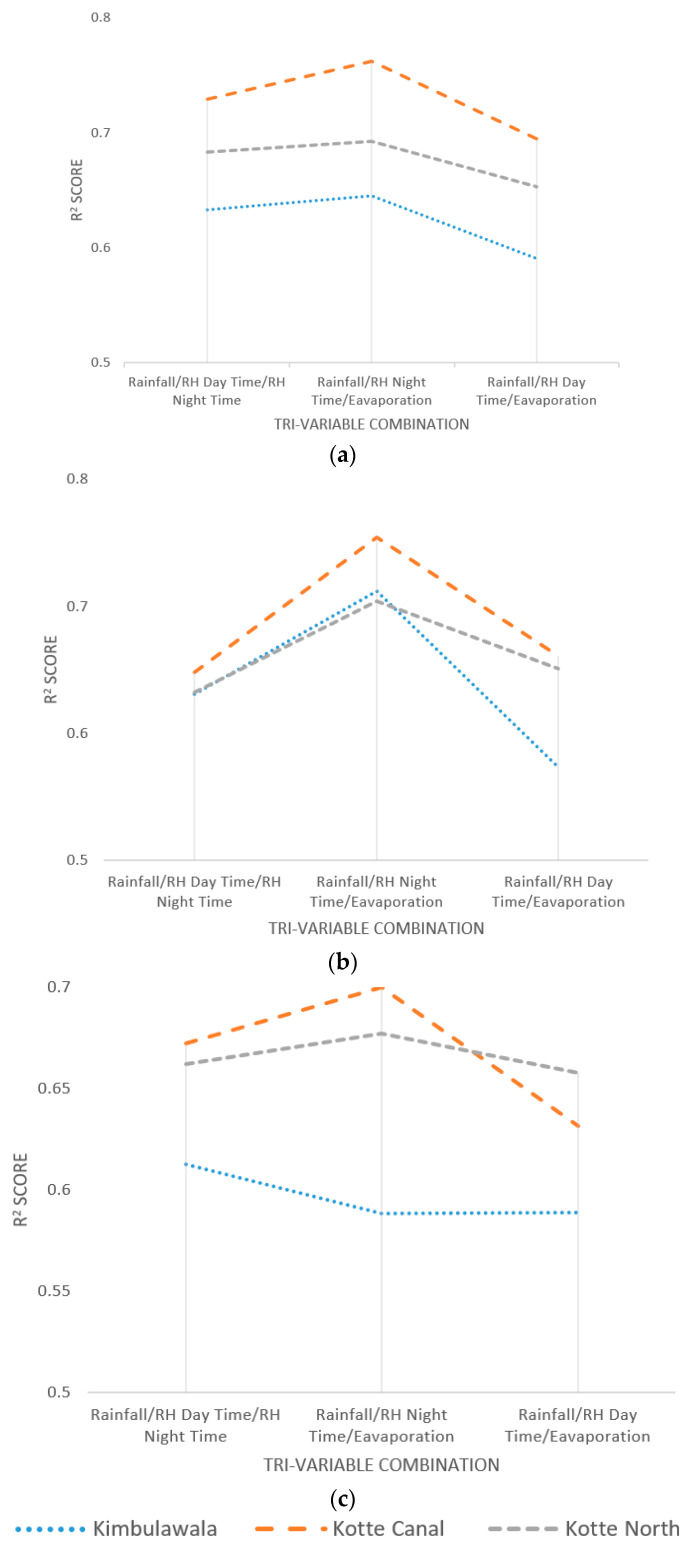
Tri-Variable Sensitivity Analysis (R^2^ Score): (**a**) under the Levenberg Marquardt Algorithm; (**b**) under the Bayesian Regularization Algorithm; (**c**) under the Scaled Conjugate Gradient Algorithm.

As per the illustration of sensitivity variation in Figure 6a–c, all the wetland data (i.e., tri-variable combination sensitivity) behave in a same way for all optimization algorithms, except the Kimbulawala wetland for the Scaled Conjugate Gradient algorithm. This confirms there was not any bias of the algorithms toward specific wetlands. Moreover, the similar behavior of all other wetlands concludes the generalization of the findings.

**Figure 7:**
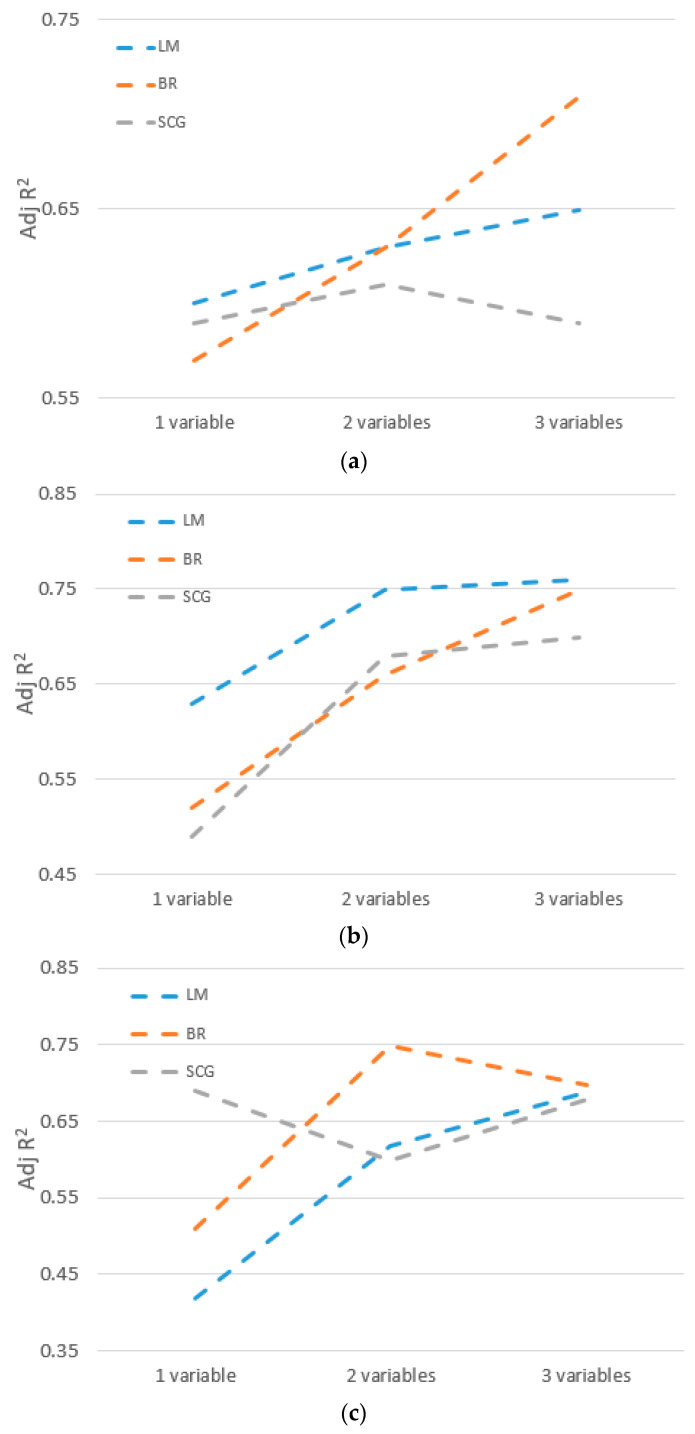
Adjusted R^2^ values: (**a**) for Kimbulawala; (**b**) for Kotte canal; (**c**) for Kotte North.

Figure 7 shows the results from the adjusted R^2^ values carried out under different variable conditions. It can be clearly seen that the Adj. R^2^ values (in most of the cases) increase with the number of independent variables (from 1 to 3). Therefore, this observation justifies the results obtained from the above sensitivity analysis.

### 4.4. Performance Comparison to Literature Found Models

Martins et al. [45] developed a deep neural network model to map wetlands using WorldView-3 and airborne LiDAR data. They used principal component analysis to identify the most important variables in wetland classification. This process was robust in optimizing the machine learning process. However, they did not discuss the water level of wetlands or investigate the most sensitive environmental parameters. Wetland water areas were predicted using neural networks by Günen [46] and Karimi [47]. However, none of these studies looked at the importance of parameters that impact the water levels and their non-linear relationships with respect to the machine learning principles.

Choi et al. [20] carried out research related to wetland water level prediction using machine learning techniques. Ss per their discussion, the unavailability of data required to train the models is one of the major obstacles to accurate prediction. They used meteorological data and upstream water level gauge data to train the water level prediction model. However, out of the selected parameters, they did not check for the most sensitive parameters that impact the water levels. Therefore, the research presented herein shows its novelty in the application. The techniques used in this paper can be used to model any other wetlands in the world, and then to understand the most important parameters impacting the water levels.

### 4.5. Future Directions of Planning and Management in the Context of Sri Lanka

Water resource management is significantly important to the capital of Sri Lanka. The city receives higher rainfalls during the months of May and June every year and floods are quite often. The flood damage in the capital city is significantly increased due to several reasons including urbanization, blocking of existing water flows, and climate change. Every year, the city experiences flood disasters at a higher cost (sometimes including lives). The wetlands consider in this analysis are the base containers of flood water to mitigate the damages. Therefore, the findings of this research work are highly important for better management of wetlands. The water levels can be effectively used to forecast the capacities of wetlands and then to divert any additional surface runoff during stormy days. This reduces the flood impacts in urbanized areas and mitigates the damages. Sustainable urban drainage systems can effectively be used to mitigate these inconveniences for the people who live and travel in the city. In addition, during the dry period, wetland water levels were observed to be decreased and that can impact the ecosystem. Therefore, having a balance of the water cycle and protecting the wetland environment are the two utmost important factors. Therefore, the findings of this research can effectively be used by the Central Environmental Authority for the sustainability of the wetlands.

## 5. Conclusions

The modelling approach used in this study is useful for determining the impact of hydro-climatic processes on wetland water levels and is a useful tool for determining the impact of climatic and hydrologic processes on wetland water levels. The method necessitates the use of basic climatic and hydrologic data, which can be found for wetland systems. In the event of unavailable meteorological data, the climate models can be successfully used for the modelling process. As a result, this method is easily applicable to other wetlands for water level simulation. The following important findings can be stated as the conclusions of this study.

- As expected, the most sensitive independent variable for wetland water level estimation is rainfall.
- Among the possible bi-variable combinations, rainfall and nighttime relative humidity presented the highest sensitivity in wetland water level estimation.
- The most sensitive tri-variable combination in the estimation of wetland water level is rainfall/relative humidity and nighttime/evaporation.
- On-site humidity meters can be used to enhance the model accuracy.

In addition, there is a considerable difference in the behavior of variables, both individual and combined. It is necessary to conduct a detailed analysis of the combined behavior of selected independent variables for wetland water level prediction for generic conclusions. At the moment, it is somewhat difficult to understand the independence and correlated behavior of variables. However, the computational efficiency can be increased significantly if the least sensitive variables can be removed from the modelling. Therefore, this study supports finding the most sensitive variable selection, which can be used to define the relative importance, the data collection frequency, and the required accuracy level. Ultimately it will support optimizing the data collection in the most cost-effective way.

In addition, low-cost sensors installed around the wetlands can be effectively used to enhance the model performance. Furthermore, climate models can be used with the appropriate bias corrections to enhance the data scare environments [48]. However, the process has to be carefully implemented with the relevant climate models.

## Figures and Tables

**Table 1:** Sensitivity (R^2^ and MSE values) of independent variable in predicting water level.

Wetland	Optimization Algorithm	Performance Index	Independent Variable						
			Rainfall	Evaporation	Wins Speed	Maximum Temperature	Minimum Temperature	Relative Humidity Day Time	Relative Humidity Nighttime
Kimbulawala	Levenberg Marquardt	MSE	0.05	0.06	0.06	0.06	0.07	0.07	0.04
		R^2^	0.60	0.34	0.47	0.15	0.35	0.46	0.61
	Bayesian Regularization	MSE	0.05	0.05	0.09	0.09	0.68	0.05	0.06
		R^2^	0.57	0.33	0.28	0.30	0.32	0.50	0.59
	Scaled Conjugate	MSE	0.05	0.06	0.06	0.07	0.07	0.06	0.06
		R^2^	0.59	0.39	0.32	0.35	0.36	0.41	0.42
Kotte Canal	Levenberg Marquardt	MSE	0.02	0.03	0.04	0.04	0.04	0.03	0.02
		R^2^	0.63	0.39	0.44	0.32	0.42	0.47	0.66
	Bayesian Regularization	MSE	0.03	0.02	0.03	0.04	0.05	0.03	0.03
		R^2^	0.52	0.44	0.42	0.36	0.39	0.55	0.60
	Scaled Conjugate	MSE	0.02	0.04	0.04	0.03	0.05	0.03	0.03
		R^2^	0.49	0.40	0.24	0.42	0.32	0.51	0.53
Kotte North	Levenberg Marquardt	MSE	0.05	0.04	0.02	0.02	0.02	0.02	0.02
		R^2^	0.42	0.51	0.38	0.25	0.31	0.49	0.58
	Bayesian Regularization	MSE	0.10	0.01	0.02	0.02	0.02	0.04	0.02
		R^2^	0.51	0.65	0.37	0.18	0.36	0.45	0.48
	Scaled Conjugate	MSE	0.03	0.02	0.10	0.06	0.06	0.95	0.08
		R^2^	0.69	0.39	0.11	0.21	0.24	0.59	0.50

**Table 2:** Results of bi-variable sensitivity analysis.

Wetland	Optimization Algorithm	Performance Index	Bi-Variable Combinations Sensitivity Analysis					
			Rainfall\Relative Humidity Nighttime	Rainfall\Relative Humidity Day Time	Rainfall\Evaporation	Relative Humidity Day Time\Night	Relative Humidity Nighttime\Evaporation	Relative Humidity Day Time\Evaporation
Kimbulawala	Levenberg Marquardt	MSE	0.04	0.06	0.04	0.05	0.05	0.08
		R^2^	0.63	0.54	0.64	0.59	0.55	0.41
	Bayesian Regularization	MSE	0.05	0.04	0.05	0.05	0.06	0.06
		R^2^	0.63	0.64	0.50	0.58	0.57	0.35
	Scaled Conjugate	MSE	0.05	0.06	0.05	0.04	0.54	0.07
		R^2^	0.61	0.62	0.54	0.55	0.49	0.44
Kotte Canal	Levenberg Marquardt	MSE	0.02	0.02	0.04	0.05	0.20	0.03
		R^2^	0.75	0.61	0.67	0.50	0.60	0.59
	Bayesian Regularization	MSE	0.02	0.03	0.04	0.03	0.04	0.05
		R^2^	0.66	0.58	0.71	0.61	0.61	0.46
	Scaled Conjugate	MSE	0.04	0.03	0.28	0.03	0.03	0.05
		R^2^	0.68	0.61	0.64	0.58	0.60	0.38
Kotte North	Levenberg Marquardt	MSE	0.01	0.01	0.04	0.01	0.55	0.02
		R^2^	0.62	0.69	0.54	0.55	0.55	0.58
	Bayesian Regularization	MSE	0.01	0.01	0.02	0.04	0.07	0.02
		R^2^	0.75	0.68	0.61	0.51	0.52	0.52
	Scaled Conjugate	MSE	0.02	0.01	0.02	0.05	0.02	0.01
		R^2^	0.60	0.61	0.62	0.51	0.56	0.54

**Table 3:** Results of tri-variable sensitivity analysis.

Wetland	Optimization Algorithm	Performance Index	Tri-Variable Combinations Sensitivity Analysis		
			Rainfall RH Day Time RH Nighttime	Rainfall RH Nighttime Evaporation	Rainfall RH Day Time Evaporation
Kimbulawala	Levenberg Marquardt	MSE	0.05	0.05	0.04
		R^2^	0.63	0.65	0.59
	Bayesian Regularization	MSE	0.05	0.03	0.05
		R^2^	0.63	0.71	0.57
	Scaled Conjugate	MSE	0.05	0.05	0.05
		R^2^	0.61	0.59	0.59
Kotte Canal	Levenberg Marquardt	MSE	0.02	0.02	0.02
		R^2^	0.73	0.76	0.69
	Bayesian Regularization	MSE	0.02	0.02	0.02
		R^2^	0.65	0.75	0.66
	Scaled Conjugate	MSE	0.03	0.02	0.02
		R^2^	0.67	0.70	0.63
Kotte North	Levenberg Marquardt	MSE	0.02	0.02	0.02
		R^2^	0.68	0.69	0.65
	Bayesian Regularization	MSE	0.02	0.01	0.02
		R^2^	0.63	0.70	0.65
	Scaled Conjugate	MSE	0.02	0.01	0.02
		R^2^	0.66	0.68	0.66

## Data Availability

The meteorological data and water level data used in this research work can be requested from the corresponding authors only for research purposes.
